# Family-based treatment with metacognitive therapy for adolescents with anorexia nervosa: findings from a phase II trial

**DOI:** 10.1186/s40337-025-01272-4

**Published:** 2025-05-26

**Authors:** Beate Østgård, Gunn Julie D. Nordahl, Arve Strandheim, Hans M. Nordahl

**Affiliations:** 1 Department of Child and Adolescent Psychiatry, St. Olavs University Hospital, Trondheim, Norway; 2 Child and Adolescent Clinic, Levanger Hospital, Nord-Trøndelag Health Trust, Levanger, Norway; 3 Department of Mental Health, Faculty of Medicine and Health, NTNU, Trondheim, Norway; 4 Department of Acute Psychiatry, Division of Mental Health Care, St. Olavs Hospital, Trondheim, Norway

**Keywords:** Anorexia nervosa, Adolescents, Metacognitive therapy, Family-based treatment, Phase II

## Abstract

**Background:**

The incidence of anorexia nervosa (AN) has increased over the past decade, particularly among school-aged children. Family-based therapy (FBT), which focuses on parental management and control, is the recommended and standard treatment for adolescents with AN. While FBT promotes weight gain and improved parent‒child interactions, it does not directly address the adolescents’ cognitive change, which could make them vulnerable to relapse. Metacognitive therapy is scarcely studied in AN, and this trial tested its feasibility and long-term effects for adolescents with AN.

**Methods:**

This phase II trial included 14 adolescents with AN in an A‒B design. Medical tests and diagnostic assessments were conducted before and after treatment. Assessments were conducted before treatment, by end of treatment and at 12 months after end of treatment. We integrated family-based treatment and metacognitive therapy for patient intervention and parental guidance.

**Results:**

At posttreatment, 13 of the 14 patients no longer met the diagnostic criteria for AN. There were significant increases in percentage expected body weight and reductions in emotional and cognitive symptoms. One patient continued to have eating disorder symptoms at posttreatment and withdrew before follow-up. No significant changes were observed in areas of family conflict or parent‒child communication. The treatment was well tolerated and deemed meaningful by both parents and patients. No adverse events or rehospitalization occurred during the 12 month follow-up period. Data collection was challenging, especially with respect to patients’ disclosure of family interactions and symptoms.

**Conclusions:**

The trial revealed positive effects posttreatment at the 12 month follow-up for 13 of 14 participants with AN. The treatment was well tolerated with no adverse effects. Despite the small sample size and lack of control, the results suggest that larger-scale trials of this intervention are warranted.

**Trial registration:**

The study has been approved as a clinical and quality audit of the delivered clinical service registered by the institutional research board at Levanger Hospital HF (IRB-13672).

**Supplementary Information:**

The online version contains supplementary material available at 10.1186/s40337-025-01272-4.

## Abstract (plain language summary)

Family-based therapy (FBT) is a recommended treatment for anorexia nervosa (AN) and focuses on parents managing the child’s meals and nourishment. While FBT helps with weight gain and improves parent‒child interactions, it does not directly address patients’ anorectic thinking, which can lead to relapse. Metacognitive therapy might help patients with emotional regulation and the underlying cognitive processes. We tested whether metacognitive therapy, combined with FBT, is effective, tolerable, and safe for adolescents with AN and their parents. The trial included 14 patients and parents. The patients were assessed before and by the end of treatment and again 12 months by end of treatment. After treatment, 13 out of 14 patients no longer met the criteria for AN. They gained weight and had fewer emotional and cognitive symptoms. One patient had AN posttreatment and left the study before the 12 month follow-up. There were no reported changes in family conflict or communication, but the treatment was well tolerated, with no adverse events or rehospitalizations. However, this study had a small sample size and no matched controls, and the results suggest that larger comparative trials should be conducted with this approach.

**Supplementary Information:**

The online version contains supplementary material available at 10.1186/s40337-025-01272-4.

## Background

Anorexia nervosa (AN) is a challenging disorder that primarily affects children and young people, often starting in early adolescence. Eating disorders present significant difficulties not only for the patients themselves but also for their parents, the social network and the health care system. Moreover, the number of patients has increased in recent years, particularly following the COVID-19 epidemic, and consequently, more individuals are now being referred for treatment in child and adolescent psychiatry. According to the Norwegian Institute of Public Health [1], the prevalence of AN increased by 67% in the age group of 6–12 years and increased by127% in the age group of 13–16 years during the COVID-19 period (2019–2021) compared with the number before the pandemic (2017–2019). In Norway, the prevalence of AN in adolescent girls was estimated to be 2.7% in 2023 [2].

Eating disorders most commonly emerge during adolescence, with the largest affected group falling between the ages of 12 and 18 years [3]. In addition to pathological underweight, a core symptom is denial and fears or anxiety related to food and meal situations, coupled with extensive concern about weight gain and bodily changes. The avoidance of food and meals exacerbates symptoms, diminishes self-control, and strains relationships with parents and siblings. Additionally, it may have serious medical effects on developmental delays, impairing endocrine disturbances such as amenorrhea and bone density loss [4]. Patients often exhibit ambivalence, emotional turmoil, and increasingly negative self-perceptions regarding their bodies.

Family-based therapy (FBT) is currently recognized as the first-hand recommended treatment for adolescents with anorexia nervosa (AN) and is widely implemented [5]. Developed within the Maudsley Model, FBT is specifically designed for adolescent outpatients with AN, with the primary goal of empowering parents to assist their children in managing eating disorder, with a focus on parental involvement and control over food and meals. [6, 7].

Several studies have reported that FBT effectively leads to weight restoration posttreatment, but relapse within the first year after treatment remains a significant risk for patients with AN, regardless of the therapy form, with occurrences reported between 6 and 17 months posttreatment [9–11]. Systematic reviews of AN studies also highlight the prevalence of posttreatment relapse [12], with relapse rates ranging from 15 to 52%, which typically increase with longer follow-up periods. In daily clinical practice, this value is likely greater.

The results of FBT indicate that parents gain increased control over their children’s eating habits, which supports patients in achieving a normal weight range posttreatment. In a comparative study of FBT and adolescent based individual therapy, FBT showed good outcomes of treating the adolescent AN by end of treatment from 42% based on PEBW > 85% and reliably improved scores on EDE. At 12 months follow-up remission rates had increased to 49% [13]. Later on Coutierer et al. [8] conducted a meta analysis of FBT compared to adolescent based individual therapy in adolescents and reported that number of patients with clinically improved body weight (PEBW > 85%) ranges between 34 and 89% and the remission rates at 6–12 months follow-up were better than for the adolescent individual based therapy [8]. Also more recently, Oshukova et al. [14] did a naturalistic study including 55 female patients receiving FBT with individual therapy reported that 61.5% of the participants achieved full weight restoration by the end of treatment, which was considered a key indicator of remission, and 88% benefitted from the treatment. This study did not provide specific relapse rates at 12-month follow-up, but it showed that participants who achieved full weight restoration at the end of therapy had better long-term outcomes.

Although there is strong evidence of the efficacy of FBT for adolescent AN by the end of the treatment, there is a high risk of relapse by the first 12 months of follow-up. An important question about the long-term outcome could be that there is little direct focus on cognitive change and processes in FBT, although it is reported that cognitive improvement occurs in successful FBT [13]. We would argue that challenging the adolescents’ cognitions related to body image and eating concerns, could be important for altering food intake and regulating emotional distress and avoidance. Helping patients to cope with negative thoughts and eating-related worries might increase their capacity to maintain weight gain and normalize their thinking and beliefs related to the body and calories after FBT.

Meta-analyses indicate that metacognitive therapy (MCT) is an effective treatment for psychological disorders [15, 16], aiming to increase cognitive flexibility and improve the metacognitive regulation of thoughts and emotions [17]. This therapy is increasingly applied to children and adolescents with anxiety, obsessive disorders, and trauma [18]. Through MCT, patients learn to better manage their overthinking by reducing body scanning, alleviating persistent worry about weight and calories, and regulating ruminations about loss of control, self-esteem and failure. It is clear how certain metacognitions are related to a drive for thinness, where beliefs about worry as a coping strategy are important factors in AN [19].

A study of 74 adult females reported that patients with AN could be characterized by heightened self-focused attention, dysfunctional beliefs about worry, and unhelpful attempts at worry control [20], which provides a good case for testing MCT in a framework of family-based treatment. In this intervention, a family-based approach is used to coach parents to effectively manage and support their child, and MCT aims at the child's ability to regulate unhelpful anorexic cognitions and behaviors related to eating.

This is the first time that MCT has been applied in a framework of the family-based approach for patients with AN. We conducted a feasibility study for integrated FBT-MCT treatment. The goal of the study was to evaluate the treatment efficacy and relapse rates for anorexia nervosa using MCT as an adjunct intervention to family-based treatment. The key areas explored in this phase II study include treatment effects; patient tolerability, including adverse events, retention rates, and relapse rates during the first-year posttreatment; and the sustainability of effects at 12 months. Although exploratory, our study was extended to evaluate the long-term effects of the treatment and relapse rates, as this is an important target in the treatment. Thus, based on earlier experiences with this approach, we expected it would be efficacious but also that addressing the cognitive changes of the patients directly would yield better long-term outcome effects and reduce relapse rates than the previously reported outcome of family-based treatments [8].

## Methods

### Design

We followed a pre-post comparative design (A-B design) involving both patients and their parents. All participants received the same treatment, and we assessed symptoms and functioning at various time points: pretreatment, during treatment (peri), by end of treatment (post) as well as at 12 months posttreatment (follow-up). To prevent immediate hospitalization, particularly for patients with critically low weights at referrals, no patients were placed on waiting lists or allocated to other unspecified treatment conditions. All eligible patients were invited to participate. Outpatient treatment was provided to all participants, combining a family-based approach and metacognitive therapy (MCT). All participants provided written consent before assessment and treatment initiation.

### Participants

This study recruited 18 adolescents with Anorexia Nervosa (F 50.0) or Atypical Anorexia Nervosa (F 50.1) aged 12 to 17 years and their families between 2016 and 2018 from an outpatient public child and adolescent mental health service (BUP) that serves a large geographic county in Middle Norway. Three eligible subjects were removed from the study before the treatment commenced; one participant was dismissed because they did not fulfill the diagnostic criteria, and 14 patients provided consent to participate and were included. The mean age was 14.4 years (SD = 1.74), and 13 girls and 1 boy completed treatment. The parents were socioeconomically middle class, and 11 parents lived together, while 3 pairs of parents lived separately. One father dropped out after the premeasuring, and one father did not participate at all. Siblings were invited but did not attend the sessions in any large degree, as school and kindergarten were prioritized by the parents.

### Assessments

Diagnostic assessments were conducted via the International Classification of Disease (ICD-10) (WHO, 1992) based on information gained from the participants, parents, general practitioners and clinical interviews. The diagnostic status of eating disorders before treatment was as follows: 11 patients (78%) were diagnosed with anorexia nervosa F50.0, and 3 patients (22%) were diagnosed with atypical anorexia nervosa F50.1.

The inclusion criteria were as follows: age; primary diagnosis of F 50.0 Anorexia Nervosa or F 50.1 Atypical Anorexia Nervosa. The patient should be treated on an outpatient basis.

The exclusion criteria were as follows: Patients who required acute hospitalization, or patients with psychosis, intellectual disabilities or other developmental disorders, Asperger syndrome, bipolar disorder, extreme self-harm or acute suicidality, or patients who could not understand or speak Norwegian.

### Measures

The primary measures used were percentage expected body weight (PEBW), and ICD-10 diagnostic status after treatment. The PEBW is calculated as weight × 100, divided by the weight of the 50th percentile for age and height. The pretreatment BMI score in the current group was 16.43 (SD = 1.74), and the PEBW was 85%, clearly indicating a harmful level of underweight in the group.

Patients and parents in this study were assessed by using observations, self-reports and rating scales as well as diagnostic assessments. The following self-reports were used: The SCORE 15 (systematic clinical outcome and routine evaluation) [21], which assesses crucial aspects of family dynamics and therapeutic change. It includes 15 Likert scale items and was presented to both the adolescent and each of the parents. It captures the perception of the family from each member from the age of 12 and above. There are three dimensions in SCORE 15: adaptability, overwhelmed by difficulties, and disrupted communication. The internal consistency of the measure, test–retest reliability and criterion validity are good, and the measure discriminates well between clinical and nonclinical cases [22, 23].

The Strengths and Difficulties Questionnaire (SDQ) [24] is a brief behavioral survey consisting of 25 items covering emotional problems, conduct problems, hyperactivity/inattention, and peer relationship problems. It is suitable for individuals aged up to 17 years and is widely used in child and adolescent psychiatry. The Norwegian version [25] is used as a screening instrument and has good psychometric properties [26, 27]. We used only a subscale of peer relations in the current study.

The Mood and Feelings Questionnaire (MFQ) [28] measures depressive symptoms in individuals aged between 6 and 17 years. It contains 34 items with scores ranging from 0–68 points. The informant related to each question by responding “not true”, “somewhat true” or “true”. The measurement has been found to be a reliable and valid self-report questionnaire and is widely used in children with childhood depression.

For cognitive attention syndrome (CAS1) [17], the youth version was adapted for young people by using age-appropriate wording and simplified sentences. This is a 16-item self-rating scale that assesses 4 dimensions: worry/rumination, threat monitoring, coping behavior and metacognitive beliefs. The CAS-1 was completed by both adolescents and parents and has shown satisfactory psychometric properties and discriminative validity [29].

### Procedure

The participants were admitted to the child and adolescent outpatient clinic after evaluation by the admission team. Patients and their parents were assigned to a specialized eating disorders team (ED team) based on referral information. Selected measurements were assessed before, during, and after treatment. All participants were medically examined and followed up by necessary medical observations and interventions. Anamnesis (developmental, food, family, and medical history) and diagnostic assessments were conducted before and after treatment, along with self-reported measures. Each family had two therapists: one focused on treating the patient, while the other coached the parents. At the end of each session, there was a joint family session with both therapists.

### Therapists

Two therapists in this trial have extensive training and experience in both FBT and MCT. They also worked as supervisors of the national education program in FBT. They received biweekly supervision on a regular basis by expert MCT therapist.

### Intervention

The Family based approach used in this study is grounded in the principles of the Maudsley model for adolescent anorexia nervosa [6, 7]. The key principles include recognizing the family as the primary resource for the child’s recovery and empowering parents to provide proper nutrition for their adolescent. The therapist works collaboratively with the family, aiding the parents in taking an active role in their child’s recovery. A crucial principle is to work on separating adolescents from those with symptoms of anorexia to externalize the illness and help the family unite against the illness. The treatment was structured into three phases with the following goals: the goal of phase 1 involves refeeding and weight restoration, where parents take control of their child's eating and meals. With the support and guidance of a therapist, they work towards achieving a normalized weight. The PEBW should gradually approach the normal level (90–95%). Phase 2 emphasized gradually returning the responsibility of eating from the parents back to the adolescent. The focus was on helping the adolescent return to normal activities, such as spending time with friends. The adolescent learned to improve age-appropriate activities and self-regulatory capacity to address persistent worry. Phase 3 aimed at consolidating normal adolescent development and living, fostering independence and autonomy in the planning for the future.

Metacognitive therapy (MCT) for eating disorders utilizes an adapted version of the generic MCT model [17]. In MCT the aim is to help both adolescents and their parents manage unhelpful thinking patterns such as worry and rumination. Patients use other coping strategies, such as avoidance, thought suppression, and continuous body scanning. These coping strategies are collectively known as cognitive attention syndrome (CAS) and create psychological distress and symptoms. The CAS contributes to symptoms and thinking styles in individuals with eating disorders, and worry is a perserverative style of thinking (overthinking) of school or athlete performance, nutrition or weight gain. The core of MCT is the metacognitive beliefs that initiate and sustain the CAS. Specific types of meta-beliefs are associated with AN. One type of negative metacognition includes fusion beliefs, such as “Thinking about food will make me fat” and “Thinking I am fat means I am.” Another type is beliefs about the uncontrollability of thoughts, such as “I cannot stop worrying” and “I cannot control thoughts.” Additionally, there are typically positive meta-beliefs about the advantages and benefits of thoughts, such as “I must suppress my feelings to keep control” and “I must control my thoughts to be strong.”

By addressing both types of metacognitions, individuals can learn to regulate their minds by disengaging from Cognitive Attentional Syndrome (CAS) and persistent negative thoughts. By working on awareness of metacognition and dysfunctional coping strategies such as worry, rumination, avoidance, and body scanning, adolescents will be able to postpone worry and eventually learn to leave anorectic trigger thoughts. The aim was to increase their cognitive flexibility (control over their minds) and sense of self-regulation, thereby reducing ongoing worry and depressive rumination.

In the intervention, FBT was used to coach the parents to effectively manage and support their child, and MCT was aimed at the child's ability to regulate unhelpful anorexic cognitions and behaviors related to eating. Parents were also introduced to information about AN, the principles of the treatment, and the role of worry and overthinking in AN.

Each session was conducted weekly during Phases 1 and 2 of the treatment and lasted 90 min. In Phase 3, sessions were held every 3–4 weeks. The treatment involved two therapists working concurrently: one focused on interventions for the parents, while the other worked directly with the patient. These sessions were brief, normally 40–45 min. The dominant issue in the first stage of treatment was minimizing the power of AN by negotiating the diet list; creating rules for exercise, meals and family activities; and helping parents manage and make adolescents feel more taken care of and safe. At the end of each session, a joint meeting was held with both therapists, the adolescent, and the parents. This joint session lasted approximately 45 min, aiming to assist the family in negotiating the diet plan, managing the patient’s activities and meal situations and fostering collaboration and teamwork between the adolescent and the parents. The number of sessions in the current trial ranged from 16 to 26, with a mean duration of 13.4 months.

### Statistical analysis

Recovery rates were evaluated using posttreatment ICD-10 diagnostic reassessment. For the treatment response of other measures, we employed repeated-measures ANOVA (rm-ANOVA), with time points (pretreatment, posttreatment, and 12-month follow-up) as the repeated measures factors. Effect sizes are reported using partial eta square (η^2^), with typical standards being small (0.01), medium (0.07), and large (0.15). Missing data were imputed via the expectation‒maximization method, which estimates the most likely values based on the observed data. All the statistical analyses were conducted using SPSS version 30.

## Results

The reassessment of the diagnoses was conducted by an independent psychiatrist, and at posttreatment, the diagnostic status was as follows: Of the 14 included adolescents and families, 13 patients no longer fulfilled the diagnostic criteria of the ED, and 1 patient (7%) still fulfilled the AN criteria (F 50.1). The recovery rate based on diagnostic status was high overall. However, 1 patient did not improve and decided to withdraw from the study at post treatment as she felt she did not become better. The patient’s BMI score was within the expected body weight, and her parents supported her decision. For PEBW, the posttreatment score for this adolescent was 101.41 (5.38) (Table 1).

**Table 1:** Repeated measures ANOVA from pre-treatment, post-treatment and 12 months follow-up in Anorexia Nervosa (N = 14)

Index	Pre-treatment	Post-treatment	12 months FU	F	df	*p*	Effect size ηp^2^
Weight, mean ± SD	46.20 ± 9.14	56.51 ± 7.76	57.85 ± 7.74	43.92	1.55,20.19	< 0.01	0.88
PEBW	85.55 ± 9.16	101.41 ± 5.38	101.22 ± 8.03	28.66	2, 11	< 0.01	0.69
Symptoms, mean ± SD							
MFQ	18.84 ± 14.36	10.92 ± 11.26	11.15 ± 11.04	5.33	1.1, 13.1	0.03	0.80
SDQ							
Emotional Distress	5.33 ± 2.50	3.83 ± 1.72	4.28 ± 2.92	2.33	2, 7	0.57	0.09
Peer relationships problems	1.33 ± 1.21	1.05 ± 0.89	1.57 ± 1.81	0.68	2, 7	0.52	0.18
SCORE15, mean ± SD							
Patient	24.01 ± 5.12	23.88 ± 4.91	23.10 ± 4.71	0.38	2, 7	0.65	0.04
Mother	23.33 ± 2.82	24.00 ± 5.65	24.11 ± 5.81	0.12	2, 7	0.88	0.04
Father	24.75 ± 5.70	23.37 ± 6.80	23.75 ± 6.31	0.68	2, 7	0.52	0.08
Cognitions, mean ± SD							
CAS 1:Patient	22.83 ± 10.01	8.33 ± 6.38	11.00 ± 8.10	18.56	1.2, 13.8	< 0.01	0.62
CAS 1: Mother	18.16 ± 7.33	7.25 ± 5.32	7.33 ± 4.99	16.35	1.1, 12.44	0.01	0.59
CAS 1: Father	11.87 ± 9.96	7.37 ± 6.94	8.62 ± 8.79	2.15	2, 6	0.15	0.40

PEBW, percentage of expected body weight; BMI, body mass index; CAS-1, cognitive attentional syndrome; MFQ, mood and feelings, depression; SCORE 15, family problems and communication; SDQ, strength and difficult questionnaire. ηp^2^ = partial eta square

Where conditions of the Mauchly’s test of sphericity were violated, we applied Huyn-Feldt correction

The adolescents’ level of depressive symptoms was measured by the MFQ [28], which covers the emotional, cognitive and physiological features of depression. The levels of depression significantly decreased from pretreatment to posttreatment and follow-up, F(2,10) = 27.59, *p* < 0.001, *ηp*^2^ = 0.80. The changes in the levels of distress and anxiety were not significant according to the SDQ (*p* = 0.37). Family conflicts and communication, as measured by SCORE 15 (Systematic Clinical Outcome and Routine Evaluation) [23], showed no significant changes (*p* = 0.38), but many missing item responses were observed on this measure.

**Fig. 1:**
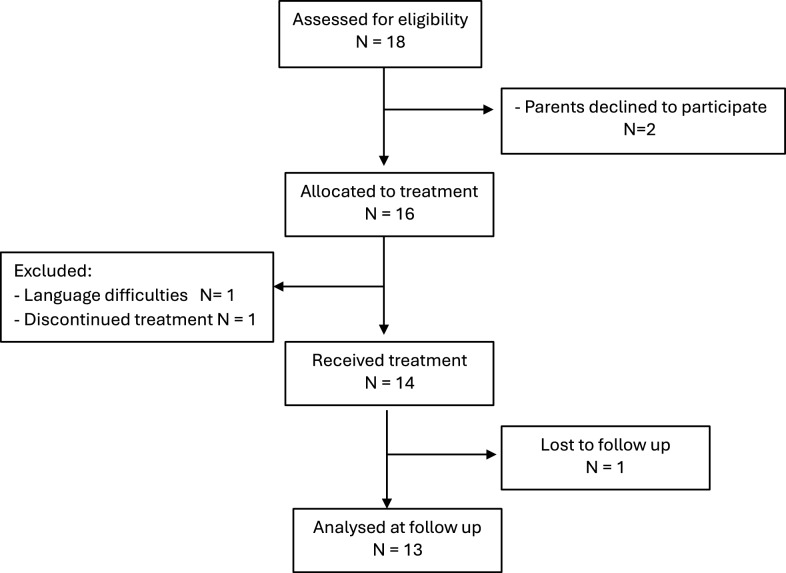
Consort flow diagram of included participants

The Cognitive Attentional Syndrome Scale (CAS-1) [29] is a measure of worry, rumination and related distress. Worry is typically related to the control of meals, calories, changes in the body and peer relationships. The ruminations are typically negative, self-analyzing and brooding not only about past failures or events but also about self-worth and the body. CAS 1 measures changes in perseverative cognitions and related distress levels. Both the patients and their parents experienced significant reductions in negative cognition, such as worry and depressive ruminations, from pretreatment to posttreatment (see Fig. 1). Moreover, the effect seemed to be significantly maintained throughout the 12-month follow-up, for the patients, F(2,10) = 21.12, *p* < 0.001, *ηp*^2^ = 0.62. Data for the eleven adolescents diagnosed with Anorexia Nervosa (F50.0) have been re-analyzed for comparative purposes (see supplementary material).

### Treatment attendance and retention

**Fig. 2:**
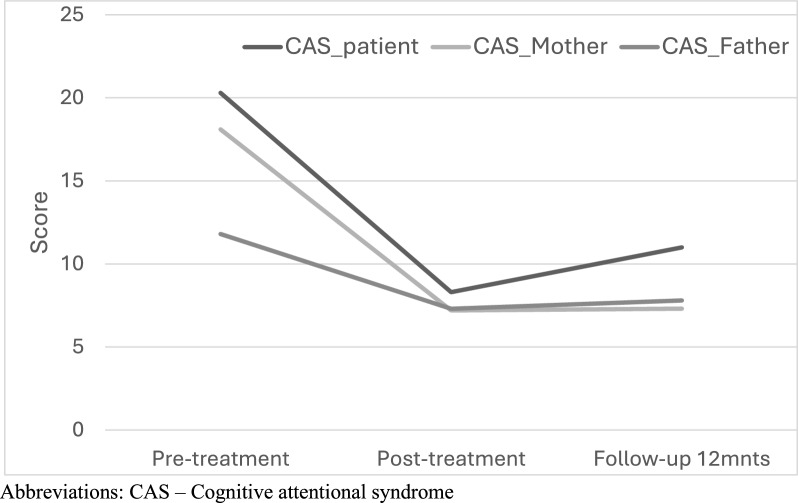
Changes in cognitive style (worry/rumination) of the patient, mothers and fathers measured by CAS 1 (N = 13)

Patients and parents attended the treatment sessions well overall. The fathers participated less frequently than the mothers did, even though they were required to attend by the therapists. However, no dropouts before or after treatment were reported. The patients completed the treatment program, except for the one formerly mentioned, and there was great variability in the number of sessions attended. The therapists followed the criteria for discharge (stable weight of PEBW and healthy autonomy of food and activities) and not a specific number of sessions (Fig. 2).

### Adverse events and tolerability

Adverse events related to patient treatment and collaboration with parents were monitored throughout the treatment phase. No adverse events were reported, and no patients were rehospitalized during the treatment or the 12 month follow-up period. However, one patient still had AN after treatment and withdrew from the study. Among the parents, one mother reported increased eating difficulties during the trial, and another set of parents developed hostility toward the therapists, ultimately refusing to attend further sessions. However, this was by the end of the treatment.

### Attrition rates

Although the measures were kept short and focused, administering and recollecting the questionnaires proved to be a challenging task. By the end of the treatment and at follow-up, the participants were not motivated to provide their responses to certain questions. Self-report measures were completed by adolescents and their parents at pre-treatment, post-treatment, and 12 month follow-up. For the SDQ, 13 out of 14 adolescents participated at pre-treatment, 10 at post-treatment, and 9 at 12 month follow-up. The MFQ had consistent participation with 13 adolescents at all three time points. For CAS 1, 14 adolescents participated at pre-treatment, 12 at post-treatment, and 12 at 12 month follow-up. The SCORE 15, assessing family dynamics and communication, had lower response rates: 14 at pre-treatment, 9 at post-treatment, and 8 at 12 month follow-up. The SCORE 15 had approximately 25% missing data and low reliability. The analysis of this measure was more underpowered than the rest, and there was limited information to derive from this measure. Additionally, it was challenging to obtain responses for the package of measures at the 12 month follow-up. Here, both participants and fathers seemed to have been missing, and we managed to obtain response from only 8 out of 14 participants on the SCORE 15. All participants received reminders written and by phone during the trial, encouraging them to return their responses. By interviewing a few parents and their children about the challenges of completing the measures provided useful information that some had a need to put things behind and disengage from the time spent in the clinic.

It seems important to personalize the invitation to participate and help the family see the usefulness of providing information. Additionally, there is a need to send polite reminders on SMS or make phone calls. In the future, incentives, such as a gift card, could be used to motivate the participants to complete the measures.

## Discussion

This preliminary study explores the enhancement of family-based treatment with metacognitive therapy for patients with AN and their parents. The study involved 14 patients and their parents, aiming to evaluate a comprehensive range of information on this combined treatment approach. The trial demonstrated that integrated family-based and metacognitive therapy, which includes both parents and patients, could be a viable and well-tolerated approach for treating AN in young individuals. All participants completed the treatment without any dropouts, but one adolescent and her parents did not complete the 12 months follow-up. The patient population was, to a large degree, underweight and had varying motivations to participate. The comorbidity rate was challenging to assess in this group, as they had several psychological and somatic complaints. Despite being seriously underweight and having low motivation to participate, the attendance and therapy completion rates were good overall.

However, taking the small sample size into consideration, we found that the patients had a significant change in percentage expected body weight at posttreatment, and the re-diagnosis revealed that all but one patient no longer fulfilled a diagnosis of an eating disorder. This is a promising result, although we need to consider that the treatment was not time-limited and continued until the patient recovered or was no longer able to continue the treatment.

The study showed a reduction in depressive symptoms as measured in the MFQ over time, along with cognitive changes as measured in the CAS-1, such as less worry and depressive rumination in both the patients and their parents. This finding indicates that patients' worry related to food, meals, the body and control has decreased. For the parents, their worry about the patient’s health also seemed to decrease, as evidenced by a decrease in worry-related distress. One exception was that when we measured the patients’ levels of distress and anxiety via the SDQ, there was no significant change. However, this result contrasted with the parents’ responses, who also completed the SDQ on behalf of their child and reported significant changes at posttreatment and follow-up (SDQ_mother_, *p* < 0.02).

We used SCORE 15 to map information about the level of family conflict and communication problems among the family members. Both the patient and the child completed this measure, and the results did not reveal any changes. We noted a lower response rate (8/14) and missing data for this measure, and it could be challenging for the child and their parents to describe how they experienced the family situation and communication with each other. We should be cautious about the interpretation of any of the results of this measure because of the high attrition of data.

The cognitive changes in worry and rumination measured by the adolescent version of the CAS-1 were significant from pretreatment to posttreatment, and the effects were maintained at the 12-month follow-up. The FBT-MCT approach may be appropriate for changing cognition in both adolescents and parents. The role of cognitions should be explored further as an indicator of maintaining weight gain over the long run, and for reductions of negative thinking styles related to performance, weight gain and body. In our experience, the frequent relapses observed in the treatment of eating disorders may primarily be due to focusing on outcomes based solely on weight gain and PEBW in adolescent AN, with little attention to obtaining cognitive changes and functioning. While more data is needed to support this contention, it remains an important hypothesis for future studies.

### Limitations of the study

There are several potential limitations to consider in the trial. First, the small number of participants reduces the statistical power and increases the risk of type II errors in the analyses, so any conclusions about efficacy should be drawn with caution. We checked the confidence intervals for PEBW and cognitive function, which were within the expected range for both measures. The outcomes of PEWB and cognitive change were significant, and the effect sizes were acceptable. Second, there was a significant amount of missing data at the 12-month follow-up, despite numerous efforts to maximize form returns through reminders via letters, phone calls, and invitations for booster sessions. The results at the 12-month follow-up were based on fewer responders, and we have limited information on the reasons of attrition. Third, although we used objective criteria for assessing outcomes and changes, better assessments of cognitions and symptoms, but also blood tests, could have provided more information about the severity of the disorder and outcomes. It should be noted that the lack of qualitative data from patients and families limits our understanding of the experiences of adolescents and their parents, particularly those who completed the treatment versus those who withdrew. Consequently, we may have missed crucial information that could help tailor interventions to the specific needs and preferences of adolescents and parents who choose to withdraw from the treatment. Fourth, we did not use the manualized FBT of adolescent anorexia nervosa, but followed the Maudsley service model, with adjustments to the original protocol. Siblings were invited and rather few were attending the sessions. These considerations should be taken into account regarding the interpretation of the results reported in this study. Finally, due to the low statistical power of the analyses, caution must be exercised when generalizing the findings to treatment effects of Family based MCT of AN.

The results of this study indicate improvements in recovery, weight gain and cognitive function. Additionally, there was indication of decreased depression post-treatment as measured by the MFQ (but not the SDQ). Notably, a substantial number of patients met the ICD-10 criteria for recovery upon reassessment. The effects on PEBW, depressive symptoms and cognitive function appeared to be sustained at the 12-month follow-up. However, we cannot rule out the possibility that these outcomes were influenced by temporal effects, as we did not include a control group.

## Conclusions

This study presents promising results for the use of family-based therapy in combination with metacognitive therapy (MCT) for anorexia nervosa, both in the short and long term. However, due to the limited scale and inherent limitations, caution is warranted in generalizing the findings. The results indicate that this treatment protocol warrants a full-scale trial, comprehensive testing, and further exploration of the significance of altering perseverative negative thinking in anorexia nervosa.

## Supplementary Information

Supplementary Material 1: Table 2 shows clinical characteristics for adolescents with diagnoses of F.50.0 Anorexia Nervosa.

## Data Availability

The data are not publicly available, because we have not asked permission from the participants. The data presented in this study are available on request from the corresponding author.

## Abbreviations

AN: Anorexia nervosa
BMI: Body mass index
BUP: Child and adolescent psychiatry unit
CAS 1: Cognitive attentional syndrome version 1
FBT: Family-based therapy
MCT: Metacognitive therapy
MFQ: Mood and feelings questionnaire
PEBW: Percentage of expected body weight
REK: Regional ethics committee
SCORE 15: Systematic clinical outcome and routine evaluation, 15 items
SDQ: Strength and difficulty questionnaire
