# Supplementary material for: Family-based treatment with metacognitive therapy for adolescents with anorexia nervosa: findings from a phase II trial
Source: J Eat Disord. 2025 May 26;13:91. doi: 10.1186/s40337-025-01272-4 (PMC12107747; doi:10.1186/s40337-025-01272-4)
Supplement: Supplementary file 1 — Supplementary Material 1: Table 2 shows clinical characteristics for adolescents with diagnoses of F.50.0 Anorexia Nervosa. [file 40337_2025_1272_MOESM1_ESM.docx]

Table 2: Repeated measures ANOVA from pre-treatment, post-treatment and 12 months follow-up in Anorexia Nervosa (F 50.0 only)

| Index | n | Pre-treatment | Post-treatment | 12 months FU | F | df | p | Effect size  ηp^2^ |
| --- | --- | --- | --- | --- | --- | --- | --- | --- |
| Weight, mean ± SD | 11 | 44.83 ± 9.36 | 56.44±8.65 | 57.46±8.40 | 40.55 | 2, 20 | < 0.01 | 0.80 |
| PEBW | 10 | 82.32 ± 6.54 | 100.81 ± 5.86 | 100.69 ± 9.01 | 32.27 | 2, 18 | < 0.01 | 0.78 |
| Symptoms, mean ± SD |  |  |  |  |  |  |  |  |
| MFQ | 10 | 16.80 ± 14.03 | 7.90 ± 6.65 | 8.51 ± 6.18 | 4.39 | 1.1, 9.7 | 0.61 | 0.32 |
| SDQ |  |  |  |  |  |  |  |  |
| Emotional Distress | 10 | 5.03 ± 2.64 | 3.61 ± 1.81 | 3.80 ± 2.38 | 0.59 | 2, 18 | 0.57 | 0.13 |
| Peer relationships problems | 9 | 1.52 ± 1.29 | 1.50 ± 0.57 | 1.75 ± 1.71 | 0.68 | 2, 16 | 0.51 | 0.17 |
| SCORE15, mean ± SD |  |  |  |  |  |  |  |  |
| Patient | 8 | 24.10 ± 5.46 | 23.50 ± 5.09 | 23.10 ± 5.04 | 0.34 | 2, 14 | 0.65 | 0.04 |
| Mother | 8 | 23.25 ± 3.01 | 24.25 ± 5.99 | 24.01 ± 6.21 | 0.15 | 2, 14 | 0.71 | 0.02 |
| Father | 7 | 26.14 ± 6.15 | 23.57 ± 6.37 | 24.71 ± 6.15 | 0.75 | 2, 12 | 0.49 | 0.11 |
| Cognitions, mean ± SD |  |  |  |  |  |  |  |  |
| CAS 1:Patient | 10 | 21.66 ± 9.87 | 7.33 ± 6.26 | 9.88 ± 7.11 | 11.54 | 1.2, 9.9 | 0.05 | 0.59 |
| CAS 1: Mother | 10 | 16.00 ± 8.68 | 6.11 ± 4.98 | 6.13 ± 4.56 | 8.26 | 1.1, 9.1 | 0.01 | 0.51 |
| CAS 1: Father | 9 | 10.20 ± 10.68 | 6.41 ± 3.84 | 8.81 ± 9.01 | 0.58 | 2, 8 | 0.58 | 0.12 |

Note: Abbreviations: PEBW: percentage of expected body weight; BMI: body mass index; CAS-1: cognitive attentional syndrome; MFQ: mood and feelings, depression; SCORE 15: family problems and communication; SDQ: strength and difficult questionnaire. ηp^2^ = partial eta square.

Note: Where conditions of the Mauchly’s test of sphericity were violated, we applied Huyn-Feldt correction.
